# Concept analysis of Caregiver Attachment: in the Context of Heart Disease

**DOI:** 10.1093/geroni/igaf122.2259

**Published:** 2025-12-31

**Authors:** Jinseon Hwang

**Affiliations:** University of Washington, Seattle, Washington, United States

## Abstract

Caregiver attachment significantly impacts patient well-being, particularly in chronic illnesses like heart disease, yet its role in caregiving remains underexplored despite extensive study in psychology. Unlike general adult attachment, caregiver attachment is shaped by illness-related stress and evolving caregiver-patient interactions, and a clearer understanding of it can enhance caregiving effectiveness, patient adherence, and emotional resilience. This study clarifies caregiver attachment in heart disease by identifying its defining attributes, antecedents, and consequences through a structured framework. A concept analysis was conducted using Walker and Avant’s (2014) eight‐step method to refine its definition in heart disease caregiving, and a literature review was performed to explore theoretical foundations and clarify conceptual boundaries. Antecedents include caregiver’s perceived responsibility, attachment history, patient dependency, relationship quality, psychological resilience, social support, and disease severity, while the four defining attributes are providing a Secure Base, Attunement, Responsiveness, and a Safe Haven. Secure caregiver attachment results in lower stress, improved adherence, and better emotional well-being, whereas insecure attachment leads to burnout, emotional detachment, and poor treatment compliance. Caregiver attachment is defined as a dynamic, emotionally attuned response fostering security, emotional stability, and mutual motivation in caregiving relationships, and recognizing it informs nursing interventions such as psychosocial support programs, caregiver education, and emotional regulation training, with future research needed to refine measurement tools and integrate attachment-based approaches into clinical practice to improve both caregiver and patient outcomes.

